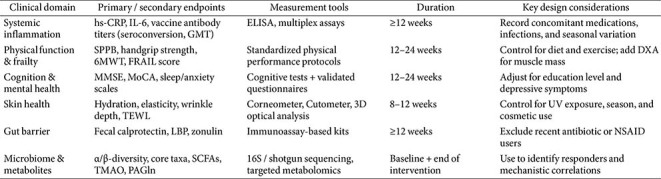# Gut Microbiota, Probiotics, and Aging: Molecular Mechanisms and Implications for Healthy Aging

**DOI:** 10.4014/jmb.2511.11046

**Published:** 2026-01-18

**Authors:** Joo-Yun Kim

**Affiliations:** R&BD Center, hy Co., Ltd., 22, Yongin-si 17086, Gyeonggi-do, Republic of Korea

**Keywords:** Gut microbiota, Aging, Probiotics, Inflammaging, Cellular senescence, Healthy aging

## Abstract

Recent advances in microbiome research have highlighted that age-related physiological changes are closely shaped by shifts in the gut microbial community rather than by the passage of time alone. Aging is frequently accompanied by a decline in microbial diversity and the loss of short-chain fatty acid-producing taxa, changes that weaken the intestinal barrier and contribute to the persistent low-grade inflammation described as inflammaging. These alterations intersect with immune and metabolic pathways linked to immunosenescence, cellular senescence, and mitochondrial function. In contrast, microbial ecosystems enriched with butyrate-producing and polyamine-generating species have been associated with more stable epithelial integrity, improved metabolic flexibility, and balanced immune activity. Emerging findings also indicate that the gut microbiota communicates with peripheral organs through the gut-skin, gut-muscle, and gut-brain axes, influencing tissue-specific aging processes. Evidence from animal models and human studies shows that dietary modulation, probiotics, and other microbiota-directed approaches can partially restore microbial functions relevant to aging, although responses vary considerably across individuals. Interest is also growing in postbiotic strategies, including microbial metabolites and vesicle-based components, which may offer targeted effects without requiring colonization. By integrating these mechanistic and translational insights, this review outlines how the gut microbiota contributes to aging biology and discusses the potential for microbiome-based interventions to support healthspan.

## Introduction

Aging is a gradual and complex biological process driven by the accumulation of molecular and cellular damage over time. This process results in the functional deterioration of organs and tissues and contributes to increased vulnerability to chronic diseases such as cardiovascular disorders, cancer, metabolic syndrome, and neurodegeneration [1]. While aging is inevitable, it is no longer viewed as entirely passive or irreversible. With global populations continuing to age, scientific focus has shifted from merely extending lifespan to enhancing healthspan, which refers to the period of life spent in good physical and cognitive function [1].

Among various factors influencing healthy aging, the gut microbiota has recently emerged as a key player. The human gastrointestinal tract harbors trillions of microorganisms that together form a complex ecosystem with critical roles in nutrient metabolism, immune modulation, and neuroendocrine communication [2-4]. Often referred to as a second genome or a crucial functional interface, the gut microbiome acts as a functional interface between the host and its environment. Disruptions to this system, commonly known as dysbiosis, have been linked to a range of age-associated conditions, including inflammation, metabolic disorders, and cognitive decline [5, 6]. Several researchers have proposed that microbial imbalance itself may represent a hallmark of aging [7], making it both a biomarker and a therapeutic target. Throughout the aging process, dynamic and reciprocal interactions occur between the host and the gut microbiota. Physiological changes such as altered diet, decreased gut motility, reduced immune surveillance, and medication use influence microbial composition, often leading to reduced diversity and a decline in beneficial taxa [2, 6, 8]. In older adults, this shift is commonly associated with a decrease in short-chain fatty acid (SCFA)-producing bacteria and an increase in pathobionts that trigger inflammation [8, 9]. These microbial alterations contribute to low-grade chronic inflammation, oxidative stress, and impaired metabolic regulation, all of which are implicated in frailty and age-related decline [8, 10, 11].

Interestingly, long-lived individuals tend to retain gut microbial profiles distinct from the general elderly population. Studies have reported enrichment of anti-inflammatory species such as *Akkermansia muciniphila*, which supports mucin degradation and SCFA production through microbial cross-feeding [12, 13]. This microbial signature may play a protective role by maintaining intestinal barrier function and reducing systemic inflammation [6, 12, 13]. However, the relevance of specific taxa can vary across populations, and inter-study discrepancies call for cautious interpretation. Notably, frail or metabolically compromised older individuals often exhibit reduced levels of *Akkermansia* and members of the *Ruminococcaceae* family, reinforcing the idea that gut microbial composition can reflect biological aging status [9]. Building upon these observational findings, experimental evidence further supports a mechanistic link between gut microbiota and aging. In support of a causal relationship, animal studies have demonstrated that transplantation of gut microbiota from young donors to aged animals improves physiological parameters and extends lifespan [14-16]. Conversely, introducing aged microbiota into young hosts induces systemic inflammation and accelerates aging-related signaling pathways. For example, young mice receiving aged microbiota show elevated levels of tumor necrosis factor-alpha (TNF-α), an effect absent in mice lacking TNF-α receptors, suggesting cytokine involvement in microbiota-induced aging [17]. Moreover, interventions such as caloric restriction and probiotic supplementation have been shown to remodel the gut microbiota toward a more youthful profile and enhance metabolic and immune function in aging models [18, 19].

Together, these findings underscore the significance of the gut microbiota as a modifiable regulator of aging. Advances in metagenomic analytics and bioinformatics have enabled the development of microbiome-based aging clocks that estimate biological age using taxonomic and functional signatures [20]. Maintaining a diverse and stable gut microbiome appears essential for controlling inflammation, preserving metabolic homeostasis, and supporting immune resilience later in life [6, 10, 18]. As research continues to illuminate the mechanisms connecting gut microbes with host aging, microbiota-targeted interventions are emerging as promising strategies to extend healthspan and improve quality of life in older populations. However, comprehensive syntheses that integrate gut-organ axes (skin, muscle, brain) with postbiotic approaches and specific aging hallmarks, including cellular senescence and inflammaging, remain limited. This review addresses this gap by bringing together molecular insights and preclinical evidence to outline a clearer framework for future personalized microbiome-based interventions.

### Gut Microbiota and Cellular Senescence

Cellular senescence is a hallmark of aging characterized by irreversible cell cycle arrest and the development of a pro-inflammatory secretory profile, known as the senescence-associated secretory phenotype (SASP) [6, 11]. Recent studies suggest that the gut microbiota plays a regulatory role in this process by modulating systemic inflammation and oxidative stress, both of which are known inducers of senescence. Age-related dysbiosis leads to increased production of reactive oxygen species (ROS) and pro-inflammatory mediators. For example, the microbial metabolite phenylacetylglutamine (PAGln) has been shown to upregulate senescence-related genes and impair cell cycle regulation in experimental models [21]. Additionally, microbial components such as lipopolysaccharides (LPS) may translocate into the bloodstream when the intestinal barrier is compromised, further promoting systemic inflammation and cellular damage [22]. Animal studies have demonstrated that young mice colonized with aged microbiota exhibit increased levels of TNF-α and elevated markers of cellular senescence. These effects were not observed in TNF-α receptor-deficient mice, indicating a cytokine-mediated mechanism [17]. Similar phenomena have been reported in invertebrate models such as *Drosophila*, where chronic microbial stimulation accelerates tissue damage and reduces lifespan through immune overactivation and ROS accumulation [23].

**Fig. 1:**
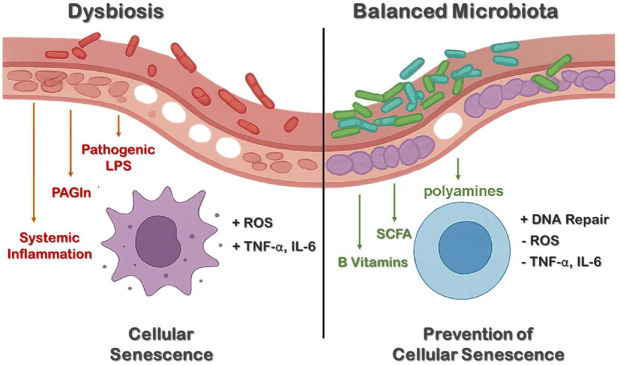
Gut microbiota and cellular senescence: inflammatory and protective pathways. The gut microbiota influences cellular senescence via inflammation and oxidative stress. Dysbiosis promotes LPS translocation, ROS accumulation, and SASP activation, leading to DNA damage and aging. Conversely, beneficial metabolites (SCFAs, polyamines, B vitamins) suppress inflammation and support cellular homeostasis by maintaining mitochondrial function and antioxidant defense.

On the other hand, several microbial metabolites appear to counteract cellular senescence. Among them, SCFAs, particularly butyrate, have been extensively studied for their anti-inflammatory and epigenetic regulatory effects. Butyrate inhibits histone deacetylases and activates cellular antioxidant pathways, contributing to the suppression of SASP gene expression in aging tissues [19]. SCFAs also maintain intestinal barrier function by serving as the primary energy source for epithelial cells and by promoting the development of regulatory T cells, thereby limiting inflammation and immune overactivation [6]. Specific probiotic strains such as *Bifidobacterium* have been shown to enhance antioxidant defenses and reduce oxidative stress in both mammalian and nematode models, with accompanying improvements in lifespan and healthspan indicators [23-25]. Moreover, gut-derived nutrients including B vitamins and polyamines support mitochondrial activity and DNA repair mechanisms, further contributing to cellular maintenance and delaying the accumulation of senescent cells [19, 26]. Taken together, these findings suggest that the gut microbiota can either accelerate or mitigate cellular senescence depending on its composition and metabolic activity. While dysbiosis promotes the accumulation of senescent cells through inflammation and oxidative stress, a balanced microbial ecosystem may activate protective pathways that preserve tissue integrity and function during aging (Fig. 1) [6, 19, 21, 23]. However, as these insights are primarily derived from preclinical models, clinical validation is required to confirm whether these specific mechanisms directly translate to human aging.

### Gut Microbiota, Inflammaging, and Immunosenescence

One of the defining biological features of aging is chronic, low-grade systemic inflammation, often referred to as inflammaging [11]. This persistent inflammatory state is characterized by elevated circulating levels of pro-inflammatory cytokines such as interleukin-6 (IL-6) and TNF-α, accompanied by a gradual decline in both innate and adaptive immune responses. This phenomenon, termed immunosenescence, contributes to impaired pathogen defense, reduced vaccine efficacy, and increased susceptibility to infections and chronic diseases in older adults [11, 27, 28]. Emerging evidence indicates that the gut microbiota plays a central role in the development and progression of both inflammaging and immunosenescence [6, 11, 22]. In healthy younger individuals, a diverse and balanced gut microbiome supports immune homeostasis through multiple mechanisms. Beneficial microbes enhance epithelial barrier integrity, stimulate the production of antimicrobial peptides, and promote the development of regulatory T cells that suppress excessive immune activation [6, 22]. However, as the microbiota becomes imbalanced with age, beneficial taxa such as *Bifidobacterium* and SCFA-producing bacteria decline, while pro-inflammatory or opportunistic species expand [6, 9, 22]. This compositional shift contributes to increased intestinal permeability and allows microbial components like LPS to enter the bloodstream. The resulting systemic exposure to endotoxins leads to chronic immune activation and sustained inflammation [22].

Animal studies provide strong causal evidence for the role of gut microbiota in driving inflammaging. In one model, transplantation of aged microbiota into young germ-free mice increased systemic levels of inflammatory cytokines and activated immune cells. These effects were not observed in TNF-α receptor-deficient animals, suggesting a critical role for cytokine-mediated signaling in microbiota-driven inflammation [17]. Human data support this association as well. Elderly individuals with microbial profiles rich in mucosa-damaging species often exhibit signs of intestinal micro-inflammation and impaired immune regulation [6, 22].

As immunosenescence progresses, the functional capacity of immune cells deteriorates. T cell diversity decreases, B cell function becomes impaired, and innate immune cells show altered activation profiles. These changes not only weaken host defense mechanisms but also reduce the effectiveness of vaccinations [11, 27, 28]. Declining levels of beneficial bacteria further exacerbate immune dysfunction. For example, *Bifidobacterium* species are known to enhance mucosal immunity and stimulate antibody production. Their reduction in the elderly may contribute to the impaired humoral response commonly observed in this population [6, 28]. Some clinical trials suggest that microbiota-targeted interventions can partially reverse these age-related immune impairments. In older adults, probiotic supplementation has been shown to enhance vaccine responsiveness, reduce gut inflammation, and modulate systemic immune markers [29]. For instance, one study reported that probiotic use improved antibody titers following influenza vaccination and lowered fecal calprotectin, a marker of intestinal inflammation. However, the magnitude of these effects varies significantly among individuals, reflecting the complexity of host-microbe interactions.

This inter-individual variability highlights the need for personalized approaches that consider baseline microbial composition, dietary habits, immune status, and genetic background [18, 30]. While probiotics and prebiotics offer a promising strategy to modulate the gut environment and improve immune health, future research should aim to identify specific microbial or host factors that predict responsiveness. The ultimate goal is to develop tailored microbiome-based therapies that support immune resilience and reduce inflammaging across diverse aging populations.

### Metabolic and Energy Homeostasis in Aging

Metabolic dysfunction is a fundamental aspect of aging, closely linked to the progressive loss of energy homeostasis, insulin sensitivity, and muscle mass. As individuals grow older, basal metabolic rate tends to decline, while adiposity increases, leading to reduced metabolic flexibility and a heightened risk of chronic conditions such as type 2 diabetes and sarcopenia [9, 11]. The gut microbiota has emerged as a critical modulator of these processes through its roles in nutrient metabolism, hormone regulation, and signaling pathways associated with cellular energy balance. In younger adults, a diverse microbial community rich in SCFA-producing bacteria helps maintain glucose homeostasis and lipid metabolism [31]. These bacteria contribute to host energy regulation by supplying butyrate and other SCFAs, which serve as energy sources for colonic epithelial cells and promote the secretion of metabolic hormones such as glucagon-like peptide-1 [6]. However, aging is often associated with a decline in microbial diversity and a reduction in SCFA-producing species, contributing to impaired gut barrier function, systemic inflammation, and metabolic instability [2, 6].

Evidence from animal models suggests that interventions such as caloric restriction can reshape the gut microbiota toward a more youthful configuration, enhancing SCFA production and restoring metabolic signaling. These changes are frequently associated with improved insulin sensitivity, reduced inflammation, and, in some cases, lifespan extension [19]. Several microbial metabolites have been shown to influence key host pathways involved in energy regulation. For example, SCFAs and other microbial products can activate AMP-activated protein kinase and sirtuin 1 (SIRT1), both of which are central regulators of mitochondrial biogenesis, oxidative stress responses, and nutrient sensing [6, 32]. In aged rodents, specific strains of *Lactobacillus* have been reported to increase expression of SIRT1 and mechanistic target of rapamycin (mTOR) in the muscle, leading to enhanced mitochondrial function and improved metabolic outcomes [19]. Other studies have documented activation of the PGC-1α pathway in muscle and liver tissues following probiotic or dietary interventions, suggesting that microbial modulation may support energy metabolism through multiple mechanisms [25, 32].

Beyond energy metabolism, the gut microbiota also supports antioxidant defense and cellular maintenance. Microbial synthesis of B vitamin complex and polyamines such as spermidine has been associated with enhanced mitochondrial enzyme activity, stimulation of autophagy, and suppression of age-related metabolic decline [19, 26]. Human epidemiological studies indicate that diets rich in fiber and fermented foods are linked to improved metabolic markers and longer lifespan, although causal relationships remain under investigation. In contrast, microbial dysbiosis characterized by high levels of endotoxins like LPS has been associated with systemic inflammation, impaired insulin signaling, and the development of metabolic syndrome and sarcopenia in older adults [22, 33]. Animal studies reinforce these findings. In aged mice, depletion of gut microbiota through antibiotics worsened muscle inflammation and atrophy, whereas transplantation of microbiota from young donors improved muscle strength, fiber morphology, and mitochondrial function [32, 33].

Collectively, these observations support the view that maintaining a diverse and metabolically active gut microbiota is essential for preserving energy homeostasis during aging. By modulating host metabolic signaling pathways, supporting mitochondrial function, and reducing systemic inflammation, the gut microbiota offers a promising therapeutic target for interventions aimed at extending healthspan and preventing age-related metabolic disorders. Translating these preclinical observations to human metabolic aging remains a key challenge, and further clinical studies are needed. From a practical standpoint, dietary strategies emphasizing fiber-rich, plant-based foods and fermented products appear to be effective and accessible approaches to maintain microbial diversity and metabolic resilience in aging populations [18, 19].

### Gut-Organ Axes in Aging: Skin, Muscle, and Brain Interactions

Recent studies have revealed that the gut microbiota not only influences systemic physiology but also interacts with specific organs through dedicated communication pathways, often referred to as the gut-organ axes. Among these, the gut-skin, gut-muscle, and gut-brain axes have garnered significant attention due to their relevance in age-related changes. The gut-skin axis illustrates how microbial imbalances can contribute to skin aging by promoting systemic inflammation and compromising epithelial integrity. Aging-associated dysbiosis and intestinal permeability can increase the translocation of inflammatory mediators into circulation, which may accelerate collagen degradation and impair skin barrier function. Clinical evidence supports this relationship. In a randomized controlled trial, supplementation with *Lactobacillus plantarum* HY7714 improved skin hydration, elasticity, and wrinkle depth in middle-aged women. Mechanistic studies suggest that this strain may act both locally and systemically by enhancing gut barrier function and modulating signaling pathways involved in dermal matrix remodeling [34-36]. Beyond specific strains, recent comprehensive reviews [37, 38] highlight that the gut microbiome influences skin aging through systemic immune modulation and the production of bioactive metabolites. Gut-derived SCFAs and phenolic compounds can reach the skin circulation, where they may exert antioxidant effects and protect against UV-induced photoaging. Furthermore, maintaining a diverse gut microbiota is crucial for suppressing systemic inflammatory cytokines that degrade collagen and elastin, thereby preserving skin structural integrity [38].

The gut-muscle axis is another emerging area of research, particularly in relation to sarcopenia, which refers to the age-related loss of skeletal muscle mass and function. Gut dysbiosis has been implicated in the pathogenesis of sarcopenia by contributing to chronic inflammation, insulin resistance, and nutrient malabsorption. In preclinical models, germ-free mice exhibit impaired muscle development, while restoration of microbial diversity through fecal microbiota transplantation from young donors improves muscle fiber size, strength, and mitochondrial activity [32, 33]. Specific strains such as *L. plantarum* HY7715 have been shown to upregulate genes related to myogenesis and mitochondrial biogenesis while downregulating catabolic and inflammatory markers. Even heat-killed forms and their extracellular vesicles demonstrate beneficial effects on muscle cell differentiation and energy metabolism under inflammatory conditions [12, 33]. These findings suggest that both live and non-viable microbial products may play a role in maintaining muscle health during aging.

The gut-brain axis further highlights the systemic influence of gut microbiota, especially in the context of neurodegeneration and cognitive decline. Aging is commonly accompanied by memory impairment, mood disorders, and increased risk of neurodegenerative diseases, all of which may be influenced by gut microbial composition. Microbial metabolites such as SCFAs, tryptophan catabolites, and trimethylamine N-oxide have been implicated in modulating blood-brain barrier integrity, neuroinflammation, and neuronal function. Dysbiosis has been associated with microglial activation, increased permeability of the gut and blood-brain barriers, and altered neurotransmitter metabolism. In clinical trials, probiotic supplementation with strains such as *L. rhamnosus* GG and *Bifidobacterium breve* has demonstrated modest improvements in cognitive performance, mood, and sleep quality in elderly individuals and patients with neurodegenerative disorders [39-42]. Although the precise mechanisms remain under investigation, the bidirectional communication between the gut and brain via neural, immune, and endocrine pathways is increasingly recognized as a therapeutic target for preserving cognitive health during aging.

Together, these gut-organ axes underscore the systemic impact of the gut microbiota beyond the gastrointestinal tract. Microbial composition and function appear to influence tissue-specific aging phenotypes across the skin, muscle, and brain. Modulating the gut microbiota through targeted dietary, probiotic, or postbiotic interventions offers a novel and integrative approach to addressing age-related decline in organ systems and may contribute to extended healthspan and improved quality of life in the elderly (Table 1).

### Preclinical Evidence: Microbiome Interventions in Aging Models

Preclinical studies have provided foundational insights into the causal relationship between the gut microbiota and the biological processes of aging. Experimental models in both vertebrates and invertebrates have demonstrated that manipulating the gut microbiota can significantly alter lifespan, healthspan, and physiological function. In the African turquoise killifish, one of the shortest-lived vertebrate models, transfer of microbiota from young donors has been shown to extend lifespan and improve locomotor activity, while aged microbiota accelerates aging phenotypes in young recipients [16]. In mammalian models, germ-free or antibiotic-treated mice commonly develop features of premature aging, including systemic inflammation, oxidative stress, and immune dysfunction. These phenotypes can be reversed by recolonization with healthy microbiota, supporting the essential role of gut microbes in maintaining physiological stability [14, 15]. In progeroid mouse models, transplantation of gut microbiota from healthy donors reduced systemic inflammatory markers and improved metabolic profiles, leading to extended survival compared with controls [14]. Furthermore, targeted administration of specific beneficial taxa has also yielded promising results. Supplementation with *Akkermansia muciniphila* has been shown to improve gut barrier integrity, attenuate immune activation, and restore bile acid metabolism in aged mice [13]. In addition, engineered microbial consortia or metabolites derived from commensal bacteria have demonstrated the ability to modulate host signaling pathways associated with longevity and stress resistance in nematodes and fruit flies [19, 23]. These findings indicate that microbiota-targeted strategies may modulate biological aging through multiple mechanisms, including metabolic remodeling, immunoregulation, and maintenance of tissue integrity. Although these results are encouraging, translation to human application requires careful validation in clinical settings, particularly with respect to long-term safety, reproducibility, and efficacy. Integration of multi-omics technologies, including metagenomics, metabolomics, and transcriptomics, will be essential for identifying the specific microbial pathways that influence aging and for developing next-generation microbiome-based interventions that are mechanistically informed and clinically applicable.

### Clinical Studies and Implications for Healthy Aging

Although human evidence remains limited compared with preclinical research, a growing number of clinical studies suggest that targeting the gut microbiota can improve health outcomes in older adults [18, 39-49]. In the NU-AGE study, a one-year intervention with a Mediterranean-style diet rich in fiber and polyphenols significantly improved gut microbial diversity and reduced frailty in elderly participants. Increases in beneficial genera such as *Bifidobacterium*, *Faecalibacterium*, and *Roseburia* were accompanied by decreases in inflammatory markers including C-reactive protein and interleukin-17, along with slower decline in cognitive and physical performance [18].

**Fig. 2:**
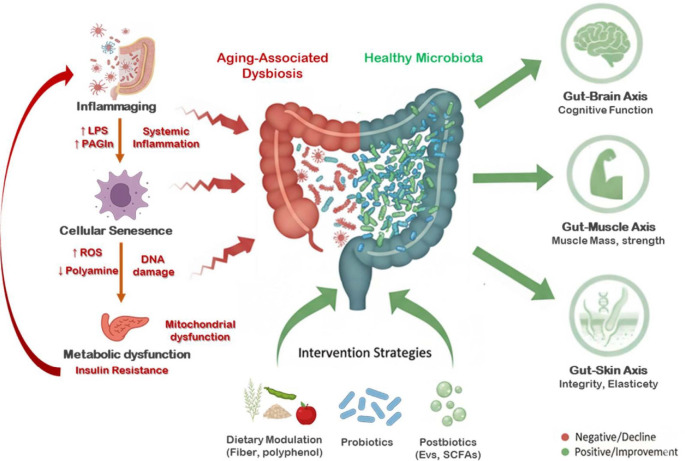
Gut microbiota, probiotics, and aging: systemic mechanisms and intervention strategies. The gut microbiota is linked to aging-related biological processes, including cellular, immune, and metabolic pathways, and influences multiple organ systems through the gut-skin, gut-muscle, and gut-brain axes. Probiotics, prebiotics, and postbiotics help restore microbial balance and support systemic mechanisms relevant to healthy aging.

Multiple probiotic trials also support beneficial effects in aging populations. Meta-analyses have shown that probiotic supplementation can reduce serum C-reactive protein levels, suggesting systemic anti-inflammatory potential [47]. In older adults, daily consumption of fermented milk containing *Lactobacillus casei* Shirota improved bowel function and gastrointestinal comfort [39]. In patients with Alzheimer’s disease, a 12-week supplementation with *Lactobacillus* and *Bifidobacterium* strains improved Mini-Mental State Examination scores and decreased oxidative stress markers [42-45]. Similarly, *L. rhamnosus* GG supplementation showed favorable effects on cognitive performance in middle-aged and older adults [32]. In Parkinson’s disease, probiotics helped alleviate constipation and were associated with modest improvements in motor function, suggesting a potential role as adjunctive therapy [40]. The systemic mechanisms and intervention strategies connecting the gut microbiota to healthy aging, including the gut-skin, gut-muscle, and gut-brain axes, are conceptually summarized in Fig. 2.

Probiotics are generally safe and well tolerated when used appropriately, although caution is advised for severely immunocompromised individuals or those with invasive medical devices [40, 46, 48, 49]. Variability among commercial products remains a concern because formulations differ in strain composition, dosage, and quality. Furthermore, inter-individual differences in gut microbial ecology, host genetics, and diet can strongly influence responsiveness to intervention [30]. These factors explain why one-size-fits-all probiotic strategies often produce inconsistent outcomes and emphasize the importance of personalized approaches that integrate baseline microbiome data, nutritional profiles, and host biomarkers [18, 30].

Despite encouraging results, clinical translation of microbiome-targeted strategies for aging presents several challenges. Heterogeneity across probiotic strains, study populations, and trial durations limits direct comparison of outcomes and complicates meta-analytical interpretation [50, 51]. Most studies have been conducted in relatively healthy older adults, but frail and complex populations that are likely to benefit most are still underrepresented [48]. Additionally, reliable biomarkers to measure biological aging or microbiome-mediated rejuvenation are still lacking. Common surrogate markers such as C-reactive protein, interleukin-6, and fecal calprotectin provide useful information on inflammation but may not accurately reflect long-term functional health outcomes [52-54]. Another barrier involves inconsistent regulatory frameworks for probiotics and live biotherapeutic products. Requirements for strain identification, manufacturing quality, quantitative labeling, and clinical claim substantiation differ substantially across regions [55-57]. To advance this field, future clinical studies should integrate multi-omics platforms, including metagenomics, metabolomics, and transcriptomics, to clarify the underlying mechanisms of probiotic or dietary effects. In addition, standardized functional outcomes including frailty indices, physical performance tests, and cognitive assessments should be adopted to ensure comparability across studies [30, 58-60]. Establishing global standards for probiotic and postbiotic classification, together with genomic verification of strains, will be essential to support responsible and reproducible clinical application of microbiome-based therapies for healthy aging [55, 57, 61]. For the standardization of these microbiome-targeted intervention studies, a framework presenting recommendations on clinical domains, endpoints, and key design considerations essential for successful clinical application is required, and an example of this is provided in Table 2.

## Conclusion

The gut microbiota plays a crucial role in modulating a wide array of biological processes that influence aging, including immune function, metabolic regulation, cellular senescence, and neurodegenerative changes [62, 63]. Growing evidence from both preclinical and clinical studies has highlighted the significant impact of gut microbes on the aging process, particularly their involvement in maintaining immune homeostasis, reducing systemic inflammation, and supporting metabolic health. Age-related dysbiosis, marked by reduced microbial diversity and a decline in beneficial bacteria, disrupts these processes, contributing to the frailty, chronic inflammation, and cognitive decline that often accompany aging. Through mechanisms such as the production of SCFAs, microbial metabolites, and the modulation of immune responses, the gut microbiota influences key aging pathways. These findings underscore the importance of maintaining a balanced and diverse microbiome as a therapeutic strategy for healthy aging. Interventions such as dietary changes, probiotics, prebiotics, and emerging postbiotics hold great promise for restoring microbial equilibrium and mitigating age-related decline in immune function, metabolic health, and cognitive capacity. Clinical studies have shown that these microbiome-targeted strategies can reduce inflammation, improve immune responses, and even enhance physical and cognitive function in older adults.

Despite these advances, significant challenges remain in fully translating these findings into clinical practice. The variability in microbiome composition across individuals, combined with the lack of standardized biomarkers for aging and microbial health, complicates the development of universally effective interventions. Additionally, differences in host genetics, diet, and lifestyle further influence the outcomes of microbiome-based therapies, necessitating personalized approaches that consider individual microbial profiles and health status. To address these limitations, future research must focus on large-scale, multi-center trials to validate efficacy, the development of standardized functional biomarkers (beyond CRP/IL-6) specifically sensitive to microbiome-mediated healthspan changes, and the utilization of AI/machine learning to predict individual responsiveness to personalized LBP or postbiotic therapy. These efforts require global harmonization of regulatory frameworks for live biotherapeutic products (LBPs) and consistent reporting standards to ensure the reproducibility and comparability of clinical outcomes. Moreover, rigorous validation of multi-omics data against physical and cognitive performance metrics is essential for translating microbial insights into tangible improvements in healthspan.

To fully translate microbial insights into actionable health strategies, a more integrated approach involving multi-omics technologies such as metagenomics, metabolomics, and transcriptomics will be essential to uncover the intricate mechanisms by which the microbiota influences aging. Combining these platforms will enable a more comprehensive understanding of how microbial communities interact with host tissues, influence signaling pathways, and contribute to functional outcomes related to immunity, metabolism, and neural health. Such integrated analyses will also help identify specific microbial signatures or metabolites that drive beneficial physiological responses, providing a stronger foundation for precision interventions. These insights will facilitate the development of targeted, personalized microbiome interventions that hold the potential to extend healthspan and improve the quality of life for aging populations. Overall, microbiome modulation is expected to become a component of strategies aimed at promoting healthy aging, supporting vitality, and helping to reduce the burden of age-associated diseases.

## Figures and Tables

**Table 1:** Summary of evidence by gut-organ axis.

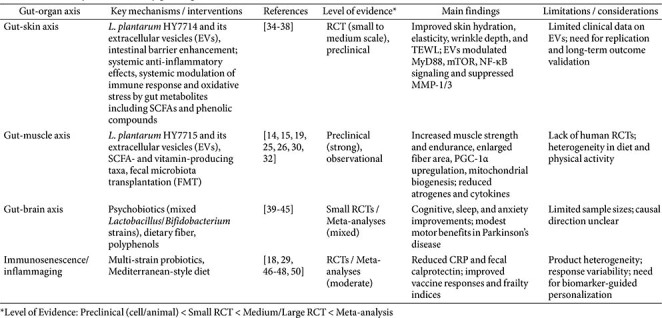

**Table 2:** Clinical framework and endpoints for human studies on microbiome-related functional outcomes.